# Transferable plasmid-mediated resistance to streptomycin in a clinical isolate of Yersinia pestis.

**DOI:** 10.3201/eid0701.010106

**Published:** 2001

**Authors:** A Guiyoule, G Gerbaud, C Buchrieser, M Galimand, L Rahalison, S Chanteau, P Courvalin, E Carniel

**Affiliations:** Laboratoire des Yersinia, Institut Pasteur, 28 rue du Dr. Roux, 75724 Paris, France.

## Abstract

Plasmid-mediated high-level resistance to multiple antibiotics was reported in a clinical isolate of Yersinia pestis in Madagascar in 1997. We describe a second Y. pestis strain with high-level resistance to streptomycin, isolated from a human case of bubonic plague in Madagascar. The resistance determinants were carried by a self-transferable plasmid that could conjugate at high frequencies to other Y. pestis isolates. The plasmid and the host bacterium were different from those previously associated with multiple-drug resistance, indicating that acquisition of resistance plasmids is occurring in this bacterial species. Emergence of resistance to streptomycin in Y. pestis represents a critical public health problem since this antibiotic is used as the first-line treatment against plague in many countries.


### Transferable Plasmid-Mediated Resistance to Streptomycin in a Clinical Isolate of *Yersinia pestis*

**Annie Guiyoule,* Guy Gerbaud,* Carmen Buchrieser,* Marc Galimand,* Lila Rahalison,† Suzanne Chanteau,† Patrice Courvalin,* and Elisabeth Carniel***
*Institut Pasteur, Paris, France; and †Institut Pasteur, Antananarivo, Madagascar

Plasmid-mediated high-level resistance to multiple antibiotics was reported in a clinical isolate of *Yersinia pestis* in Madagascar in 1997. We describe a second *Y. pestis* strain with high-level resistance to streptomycin, isolated from a human case of bubonic plague in Madagascar. The resistance determinants were carried by a self-transferable plasmid that could conjugate at high frequencies to other *Y. pestis* isolates. The plasmid and the host bacterium were different from those previously associated with multiple-drug resistance, indicating that acquisition of resistance plasmids is occurring in this bacterial species. Emergence of resistance to streptomycin in *Y. pestis* represents a critical public health problem since this antibiotic is used as the first-line treatment against plague in many countries.

*Yersinia pestis* is the causative agent of plague, a disease transmitted from rodent to rodent by the bites of fleas. Bubonic plague, the most common form of the disease, occurs through rodent-to-human transmission by infected fleas of peridomestic animals (rats, cats) or wild rodents. Pneumonic plague, a less frequent but highly severe form of the disease, is transmitted from human to human by infected droplets spread by a patient with lung infection (1).

Public health measures and effective antibiotic treatments have led to a drastic decrease in plague worldwide. However, the disease is not eradicated. Endemic plague foci persist in Africa, Asia, and North and South America. During the last 15 years (1982 to 1996), 23,904 human plague cases and 2,105 deaths were reported to the World Health Organization by 24 countries (2). The most affected countries are Madagascar and Tanzania in Africa, Vietnam in Asia, and Peru in the Americas. Since the early 1990s, a steadily increasing trend in human plague cases has been reported to the World Health Organization. This trend is partly attributable to epidemics in places where human plague had disappeared for several decades and has led the World Health Organization to categorize plague as a reemerging disease (3). The reasons for plague's reemergence are not clear but may be partly attributable to inadequate surveillance.

Address for correspondence: Elisabeth Carniel, Laboratoire des *Yersinia*, Institut Pasteur, 28 rue du Dr. Roux, 75724 Paris Cedex 15, France; fax: 33-1-40-61-30-01; e-mail: carniel2@pasteur.fr.

Streptomycin, chloramphenicol, and tetracycline, alone or in combination, are the reference drugs to treat plague, whereas tetracycline or sulfonamides are recommended for prophylaxis (4). Classically, *Y. pestis* isolates are uniformly susceptible to all antibiotics active against gram-negative bacteria (5-7). Recently, high-level resistance to multiple antibiotics, including those recommended for plague prophylaxis and therapy, was observed in a clinical isolate of *Y. pestis* in Madagascar (8).

We report high-level resistance to streptomycin (the reference antibiotic for plague treatment) in a second strain of *Y. pestis* isolated in Madagascar. The resistance genes were carried by a plasmid that could conjugate at high frequencies to other *Y. pestis* isolates.

### Materials and Methods

### Patient and Strains

In our study of the second resistant *Y. pestis* isolate, *Y. pestis* 16/95, we used the bacterial strains listed in Table 1. The isolate, biotype Orientalis and ribotype Q, was obtained in 1995 in the Ampitana District of Madagascar from the axillary bubo puncture of a 14-year-old boy before antibiotic treatment. No recent history of travel outside the village was noted. Dead rats were found inside his house before the onset of the disease. The patient was treated with twice a day intramuscular injections of streptomycin (2 g per day for 4 days) and oral trimethoprim-sulfamethoxazole (2 g per day for 10 days) and recovered.

### Media and Resistance Studies

Brain-heart infusion broth and agar (Difco Laboratories, Detroit, MI) were used. The MICs of antibiotics were determined on Mueller-Hinton agar (Sanofi Diagnostics Pasteur, Marnes-la-Coquette, France). The cultures were incubated for 18 hours at 37°C for *Escherichia coli* and for 48 hours at 28°C for *Yersinia* strains. Aminoglycoside-modifying enzymes were assayed by the phosphocellulose paper-binding technique (13) in supernatants (centrifuged at 100,000 x *g*) after ultrasonic bacterial disintegration. Mating on filters was performed as described previously (8). Transfer frequencies were expressed as the number of transconjugants per donor colony-forming unit after the mating period. Antibiotic concentrations for selection were 100 mg/L for ampicillin, 50 mg/L for nalidixic acid, 100 mg/L for rifampicin, and 25 mg/L for streptomycin.

### Nucleic Acid Techniques

Isolation of plasmid DNA, cleavage of restriction fragments, and purification of DNA fragments from agarose type VII (Sigma Chemical Co., St. Louis, MO) were performed as described elsewhere (14). Pulsed-field gel electrophoresis was performed for 18 hours with a CHEF-DRIII apparatus (Bio-Rad Laboratories, Richmond, CA), by using an electric field of 6 V/cm and an angle of 120°. Initial and final pulse times were 0.1 second and 6 seconds, respectively. Migration of the DNA fragments was performed in 0.5X Tris-Borate-EDTA buffer in a 0.9% agarose gel at 17°C. DNA sequencing reactions were performed with a Taq BigDye Terminator cycle sequencing kit (Applied Biosystems, Foster City, CA) in a Perkin-Elmer 9700 thermocycler. The samples were loaded onto 4% polyacrylamide gels and electrophoresed on a Model ABI PRISM 377 automatic DNA sequencer (Perkin-Elmer, Norwalk, CT). The nucleotide sequence of the

Table 1. Bacterial strain used in study of resistant *Yersinia pestis* isolate 16/95

| Strain | Characteristics and plasmid content[a] | Source or reference |
|---|---|---|
| ***Y. pestis*** | | |
| 16/95 | pFra, pPla, pYV, pIP1203 $Tra^{+b}$ $Sm^{c}$ | Wild strain |
| 6/69 | pFra, pPla, pYV | Wild strain |
| 6/69cN | $Nal^{d}$, pFra, pPla | Spontaneous Nal mutant of pYV cured 6/69 |
| 6/69cNR | Nal, $Rif^{e}$, pFra, pPla | Spontaneous Rif mutant of 6/69cN |
| 6/69cN(pIP1203) | Nal, pFra, pPla, pIP1203 $Tra^{+}$ Sm | Transconjugant 16/95 x 6/69cN |
| ***Y. pseudotuberculosis*** | | |
| IP32790 | pYV | Wild strain |
| IP32790cN | Nal | Spontaneous Nal mutant of pYV cured P32790 |
| IP32790cN(pIP1203) | Nal, pIP1203 $Tra^{+}$ Sm | Transconjugant 16/95 x IP32790cN |
| ***Y. enterocolitica*** | | |
| IP864 | pYV | Wild strain |
| IP864cN | Nal | Spontaneous Nal mutant of pYV cured IP864 |
| ***Escherichia coli*** | | |
| C600R | *thr leuB6 thi-1 lacY supE rpoB* | Spontaneous Rif mutant of C600, Bachmann (9) |
| JM109 | *hsdR*$^{-}$ *supE gyrA* | Yanisch-Perron et al. (10) |
| K802N | *hsdR*$^{-}$ *hsdM*$^{+}$ *gal*$^{-}$ *met*$^{-}$ *supE gyrA* | Wood (11) |
| K802N(pIP1203) | pIP1203 $Tra^{+}$ Sm | Transconjugant 16/95 x K802N |

[a]Plasmid content = pFRa, pPla, and pYV are the endogenous plasmids of *Y. pestis* (12).
[b]$Tra^{+}$ = self-transferable.
[c]Sm = streptomycin resistance.
[d]Nal = nalidixic acid resistance.
[e]Rif = rifampicin resistance.

*strA* and *strB* genes and of flanking regions from pIP1203 has been deposited in the EMBL data bank under accession number AJ249779.

### Results

### Streptomycin Resistance in *Y. pestis* 16/95

Disk-agar diffusion tests showed that *Y. pestis* 16/95 was resistant to streptomycin but remained susceptible to spectinomycin and other antibiotics, including those recommended for plague therapy (chloramphenicol and tetracycline) and prophylaxis (sulfonamides and tetracycline) (4). The MICs of streptomycin and spectinomycin for this strain were 1,024 mg/L and 16 mg/L, respectively. High-level resistance was due to the presence of a streptomycin phosphotransferase activity. No adenylyl transferase activity was found (data not shown).

### Transfer of Streptomycin Resistance to Other Bacterial Species

Attempts were made to transfer streptomycin resistance from *Y. pestis* 16/95 by conjugation to recipient strains (MIC $\leq$ 8 mg/L) (Table 2). Transfer occurred at high frequencies (3 x $10^{-1}$ per donor CFU) to *Y. pestis* (MIC = 1,024 mg/L) and *Y. pseudotuberculosis* (MIC = 256 mg/L) and at lower frequencies to *E. coli* (MIC = 128 mg/L); transfer to *Y. enterocolitica* was not detected. Retransfer of streptomycin resistance from *Y. pestis* and *Y. pseudotuberculosis* transconjugants to *Y. pestis* and *Y. pseudotuberculosis* also occurred at high frequencies (3 x $10^{-2}$) and was less efficient when *E. coli* was used as the recipient strain.

### Characterization of Plasmid pIP1203

Plasmid DNA was extracted from *Y. pestis* 6/69 and 16/95 and digested by *Eco*RV (Figure 1A).

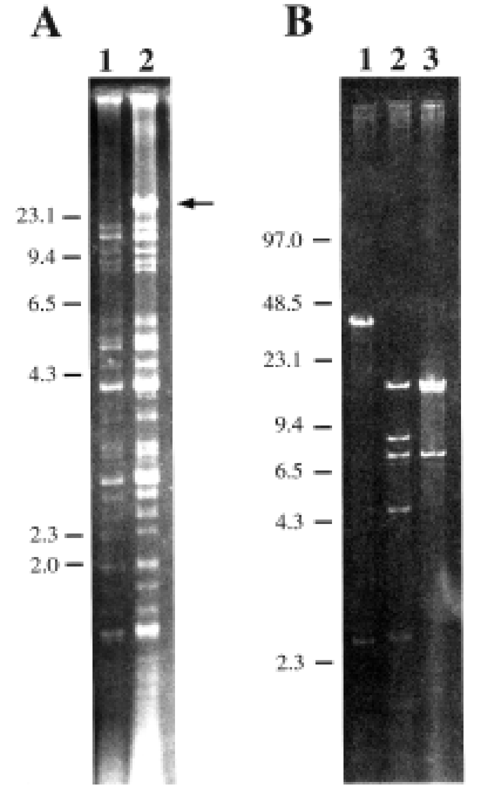

Figure 1. Analysis of plasmid pIP1203. A) Agarose-gel electrophoresis of *Eco*RV-digested plasmid DNA from representative *Yersinia pestis* strain 6/69 (1) and from streptomycin-resistant strain 16/95 (2). B) Pulsed-field gel electrophoresis of pIP1203 DNA extracted from *Escherichia coli* K802N transconjugant and digested by *Eco*RV (1), *Eco*RI+*Eco*RV (2), and *Eco*RI (3). The arrow indicates the extra large-size DNA fragment in strain 16/95. The size of the molecular weight markers in kilobases is indicated at the left of the gels.

Table 2. Conjugative transfer of pIP1203

| Donor | Recipient | Frequency of transfer |
|---|---|---|
| *Yersinia pestis* 16/95 | *Y. pestis* 6/69cN | 3 x $10^{-1}$ |
| | *Y. pseudotuberculosis* IP32790cN | 1 x $10^{0}$ |
| | *Y. enterocolitica* IP864cN | <$10^{-7}$ |
| | *E. coli* K802N | 2 x $10^{-5}$ |
| *Y. pestis* 6/69cN (pIP1203) | *Y. pestis* 6/69cNR | 2 x $10^{-1}$ |
| | *Y. pseudotuberculosis* IP32790cNR | 5 x $10^{-1}$ |
| *Y. pseudotuberculosis* IP32790cN(pIP1203) | *Y. pestis* 6/69cNR | 3 x $10^{-2}$ |
| | *Y. pseudotuberculosis* IP32790cNR | 2 x $10^{-2}$ |
| | *E. coli* C600R | <$10^{-7}$ |
| *Escherichia coli* K802N (pIP1203) | *Y. pestis* 6/69cNR | 6 x $10^{-2}$ |
| | *Y. pseudotuberculosis* IP32790cN | 1 x $10^{-3}$ |
| | *E. coli* C600R | 1 x $10^{-5}$ |

The restriction fragments in strain 6/69 correspond to those of the three *Y. pestis* resident plasmids, pPla, pYV, and pFra (12). Comparison of the restriction profiles of strains 6/69 and 16/95 revealed that the latter contained at least one extra large-size *Eco*RV fragment corresponding to an additional plasmid, designated pIP1203. Upon transfer to *E. coli*, the size of pIP1203 was estimated (after single and double digestion with *Eco*RI and *Eco*RV) to be approximately 40 kb (Figure 1B).

Plasmid pIP1203 was stable after 100 generations in *Y. pestis* 16/95 and *E. coli* K802N (frequency of loss <0.25%). In experiments performed by reciprocal conjugation to assess the incompatibility group, pIP1203 exhibited strong incompatibility with plasmid RP4 (data not shown), which belongs to the IncP group. No incompatibility with prototype plasmids of incompatibility groups Inc FI, FII, I1, I2, N, 6-C, 7-M, 10-B-O, J, T, and W was observed (15).

### Characterization of the Streptomycin-Resistance Genes

To clone the streptomycin-resistant determinant, DNA from plasmids pIP1203 and pUC18 was partially digested with *Sau*3AI and *Bam*HI, respectively, ligated, and introduced into *E. coli* JM109 (MIC of streptomycin = 2 mg/L). The smallest recombinant plasmid conferring streptomycin resistance, pAT709, contained an 11-kb insert. The resistance determinant was subcloned by introducing a 2.7-kb *Hin*cII fragment of the 11-kb insert into pUC18, which generated pAT710. This recombinant plasmid conferred high levels of resistance to the new host (MIC of streptomycin = 512 mg/L) by synthesis of a streptomycin phosphotransferase. Sequence determination of the insert in pAT710 revealed that the base composition of this fragment was 57.5% G+C, much higher than the average G+C content of the *Y. pestis* genome (46%) (16). Two potentially coding sequences of 801 bp and 834 bp identified in the insert were identical to the *strA* and *strB* genes that encode an aminoglycoside 3"-*O*-phosphotransferase [APH(3")-Ib] and a 6-*O*-phosphotransferase [APH(6)-Id], respectively (17). The *str* genes were originally described in the small, nonconjugative, broad-host-range IncQ plasmid RSF1010 (18). They have been subsequently found as part of transposon Tn*5393* and related elements in phytopathogenic *Erwinia amylovora*, *Pseudomonas syringae* pv. papulans, and *Xanthomonas campestris* pv. vesicatoria (19,20).

An 81-bp sequence identical to the inverted terminal repeat (IR) of Tn*5393* was identified downstream from pIP1203 *strA-strB* genes (Figure 2). This IR is always present at the same position in the various genetic structures that

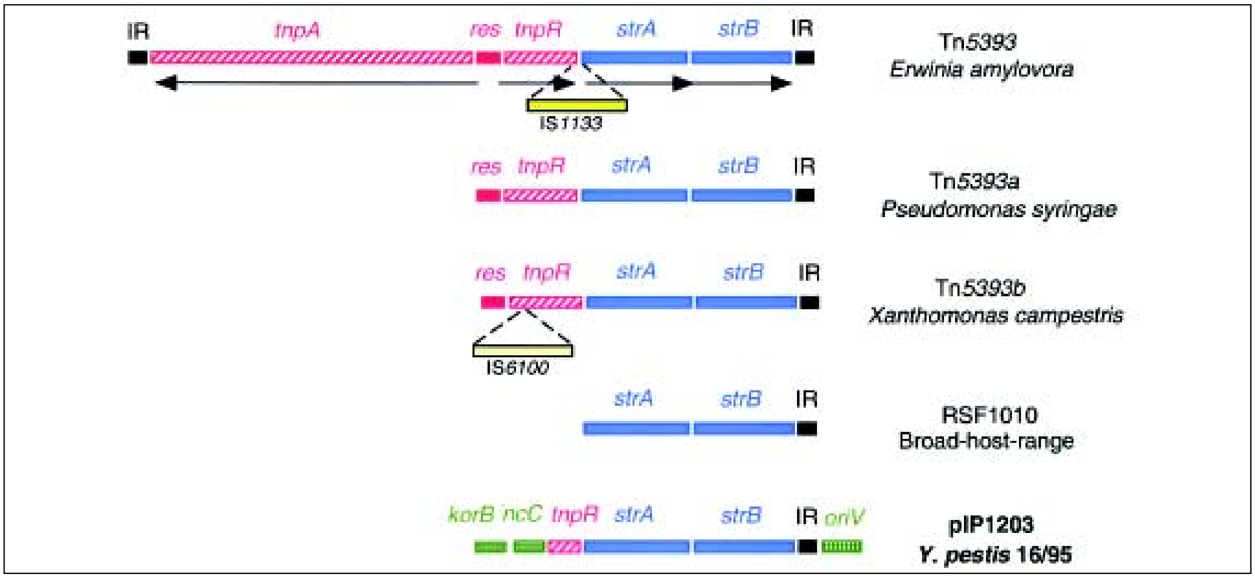

Figure 2. Genetic organization of the *strA-strB* genes. Schematic representation of the regions of Tn*5393* and derivatives and of plasmids RSF1010, and pIP1203 carrying the *strA* and *strB* genes. IR, inverted repeat; *tnpA*, transposase; *res*, resolution site; *tnpR*, resolvase; IS*1133* and IS*6100*, insertion sequences; *korB* and *incC*, genes homologous to those involved in regulation and partition of plasmid R751, respectively; *oriV*, origin of vegetative replication of R751. Direction of gene transcription is indicated by arrows.

carry the *str* genes. Upstream from *strA*, the sequence was identical to a portion of the *tnpR* resolvase-repressor gene of Tn*5393*, Tn*5393*a, and Tn*5393*b (20). The identity was interrupted after 105 bp within the 3'-end of *tnpR* (Figure 2). Like Tn*5393*a, pIP1203 possessed the TAG motif, which represents a putative insertion target for IS*1133* (20).

The *tnpR*-*strA*-*strB*-IR region of pIP1203, which displayed a Tn*5393*-like organization, was flanked on both sides by sequences highly similar to portions of the broad-host-range IncP plasmid R751 (21). Upstream from the truncated *tnpR* gene, there was identity with the 3'-end of the *incC2* and the 5'-end of the *korB* genes [positions 3396 to 3820 of plasmid R751, numbering according to GenBank accession number U67194]. Downstream from the IR, identity was found with a portion of plasmid R751 (positions 9796 to 9947, numbering according to GenBank accession number U67194), located in the vicinity of the *oriV* vegetative origin of replication.

### Discussion

*Y. pestis* strain 16/95, isolated in Madagascar in 1995 from a human case of bubonic plague, carried the self-transferable plasmid pIP1203 conferring resistance to streptomycin. The strain of *Y. pestis* 17/95 harboring the multidrug-resistance conjugative plasmid pIP1202 described in 1997 (8) was also isolated in Madagascar from a human case. However, the two strains differ in several aspects: they were isolated in two districts of Madagascar (Ambalavao and Ampitana) that are 120 km (80 miles) apart; strain 17/95 is of the typical ribotype B, whereas strain 16/95 is of the newly described, Madagascar-specific, ribotype Q (22); plasmid pIP1202 carries multiple antibiotic resistance genes, belongs to the Inc6-C group, and is 150 kb in size, whereas pIP1203 carries only the streptomycin resistance determinants, belongs to the IncP group, and is 40 kb in size; and streptomycin resistance is due to adenylylation of the drug in strain 17/95 and to phosphorylation in strain 16/95. Therefore, the two resistant *Y. pestis* isolated in Madagascar correspond to distinct strains that have acquired different conjugative plasmids.

The streptomycin resistance genes in pIP1203 are part of the *tnpR*-*strA*-*strB*-IR cluster characteristic of the Tn*5393* group of transposons. This portion of the element is inserted in R751, a broad-host-range plasmid belonging to the IncP group. The sequences flanking the *tnpR*-*strA*-*strB*-IR region in pIP1203 are separated by approximately 6 kb in the original R751 backbone (21). This organization suggests that insertion of a Tn*5393*-like element was associated with concomitant (or subsequent) loss of a region involved in the control of plasmid stability. Despite this deletion, pIP1203 appears to be highly stable in both *Y. pestis* and *E. coli*.

IncP plasmids are promiscuous; therefore, the original host of pIP1203 remains unknown. However, since this plasmid was extremely stable in *Y. pestis* 16/95, conferred high-level resistance to streptomycin, and could transfer at remarkably high frequencies to other strains of *Y. pestis*, it is possible that pIP1203 was acquired a long time ago and is now well adapted to this bacterial species.

It is not known where genetic transfer of the resistance plasmid took place. During its flea-host-flea cycle, *Y. pestis* may have been in contact with the donor cell, either in its mammalian host (rodent or human) or the insect vector. In mammals, *Y. pestis* circulates in a usually sterile milieu (lymphatic vessels, spleen, liver, blood, and sometimes lungs). Contact with the bacterial donor and transfer of pIP1203 may have occurred in the bloodstream at the premortem stage of infection, when gut bacteria invade the host. Alternatively, plasmid acquisition may have taken place in the midgut of the flea, a nonsterile environment where *Y. pestis* is most likely to be in intimate contact with other microorganisms.

From a clinical and public health point of view, this report is of great concern and indicates that surveillance of antibiotic resistance in *Y. pestis* should become systematic worldwide. Streptomycin, an inexpensive, easy to use, and highly effective drug against *Y. pestis,* represents the therapy of choice for plague in Madagascar. Spread of plasmids pIP1202 and pIP1203 among strains of *Y. pestis* would render streptomycin ineffective for plague treatment and could create economic and therapeutic problems in Madagascar and other countries with endemic plague foci.

This work was supported in part by a Bristol-Myers Squibb Unrestricted Biomedical Research Grant in Infectious Diseases.

A. Guiyoule is a research technician. Her research interests are bacterial pathogenesis and antibiotic resistance.

### References

1. Pollitzer R. Plague. In: WHO Monograph Series 22 World Health; Geneva: World Health Organization; 1954.
2. World Health Organization. Human plague in 1996. Wkly Epidemiol Rec 1998;73:366-9.
3. Schrag SJ, Wiener P. Emerging infectious diseases: what are the relative roles of ecology and evolution? Trends Evol Ecol 1995;10:319-24.
4. Barnes AM, Quan TJ. Plague. In: Gorbach SL, Bartlett JG, Blacklow NR, editors. Infectious diseases. Philadelphia: W. B. Saunders Company; 1992:1285-91.
5. Rasoamanana B, Leroy F, Raharimanana C, Chanteau S. Surveillance de la sensibilité aux antibiotiques des souches de *Yersinia pestis* à Madagascar de 1989 à 1995. Arch Inst Pasteur Madagascar 1995;62:108-10.
6. Frean JA, Arntzen L, Capper T, Bryskier A, Klugman KP. In vitro activities of 14 antibiotics against 100 human isolates of *Yersinia pestis* from a southern African plague focus. Antimicrob Agents Chemother 1996;40:2646-7.
7. Smith MD, Vinh DX, Hoa NTT, Wain J, Thung D, White NJ. In vitro antimicrobial susceptibilities of strains of *Yersinia pestis*. Antimicrob Agents Chemother 1995;39:2153-4.
8. Galimand M, Guiyoule A, Gerbaud G, Rasoamanana B, Chanteau S, Carniel E, et al. Multidrug resistance in *Yersinia pestis* mediated by a transferable plasmid. N Engl J Med 1997;337:677-80.
9. Bachman B. Derivations and genotypes of some mutant derivatives of *Escherichia coli* K-12. In: Neidhardt F, editor. *Escherichia coli* and *Salmonella*, cellular and molecular biology, 2nd ed. Washington, DC: ASM Press; 1996:2460-88.
10. Yanisch-Perron C, Vieira J, Messing J. Improved M13 phage cloning vectors and host strains: nucleotide sequences of the M13mp18 and pUC19 vectors. Gene 1985;33:103-19.
11. Wood WB. Host specificity of DNA produced by *Escherichia coli*: bacterial mutations affecting the restriction and modification of DNA. J Mol Biol 1966;16:118-33.
12. Ferber DM, Brubaker RR. Plasmids in *Yersinia pestis*. Infect Immun 1981;31:839-41.
13. Haas MJ, Dowding JE. Aminoglycoside-modifying enzymes. Methods Enzymol 1975;43:611-28.
14. Sambrook J, Fritsch EF, Maniatis T. Molecular cloning: a laboratory manual. 2nd ed. Cold Spring Harbor (NY): Cold Spring Harbor Laboratory Press; 1989.
15. DNA insertion elements, plasmids, and episomes. In: Bukhari AI, Shapiro JA, Adhya SL, editors. Cold Spring Harbor (NY): Cold Spring Harbor Laboratory Press; 1977. p. 601-38.
16. Bercovier H, Mollaret HH, Alonso JM, Brault J, Fanning GR, Steigerwalt AG, et al. Intra- and interspecies relatedness of *Yersinia pestis* by DNA hybridization and its relationship to *Yersinia pseudotuberculosis*. Curr Microbiol 1980;4:225-9.
17. Shaw KJ, Rather PN, Hare RS, Miller GH. Molecular genetics of aminoglycoside resistance genes and familial relationships of the aminoglycoside-modifying enzymes. Microbiol Rev 1993;57:138-63.
18. Scholz P, Haring V, Wittmann-Liebold B, Ashman K, Bagdasarian M, Scherzinger E. Complete nucleotide sequence and gene organization of the broad-host-range plasmid RSF1010. Gene 1989;75:271-88.
19. Chiou CS, Jones AL. Nucleotide sequence analysis of a transposon (Tn*5393*) carrying streptomycin resistance genes in *Erwinia amylovora* and other gram-negative bacteria. J Bacteriol 1993;175:732-40.
20. Sundin GW, Bender CL. Expression of *strA-strB* streptomycin resistance genes in *Pseudomonas syringae* and *Xanthomonas campestris* and characterization of IS*6100* in *X. campestris*. Appl Environ Microbiol 1995;61:2891-7.
21. Thorsted PB, Macartney DP, Akhtar P, Haines AS, Ali N, Davidson P, et al. Complete sequence of the IncPß plasmid R751: implications for evolution and organisation of the IncP backbone. J Mol Biol 1998;282:969-90.
22. Guiyoule A, Rasoamanana B, Buchrieser C, Michel P, Chanteau S, Carniel E. Recent emergence of new variants of *Yersinia pestis* in Madagascar. J Clin Microbiol 1997;35:2826-33.
